# Effect of Smoking on the Healing of a Mandibular Condyle Fracture

**Published:** 2021-03-08

**Authors:** Kun Hwang

**Affiliations:** Department of Plastic Surgery, Inha University School of Medicine, Incheon, Korea

**Keywords:** cigarette smoking, mandibular fractures, mandibular condyle, fracture fixation, immobilization

## Abstract

**Background:** We experienced a case of malunion of condylar fracture after miniplate fixation in a patient with a 40 pack-year smoking history who restarted smoking at 5 weeks postoperatively. **Case:** A 64-year-old man lost consciousness and fell down, hitting his chin on the floor. He had malocclusion and open bite bilaterally. The mouth opening was 1.5-finger breadths. He had a 40 pack-year smoking history. Radiology revealed a bilateral condylar fracture and a fracture of the parasymphysis. Intermaxillary fixation was done using the skeletal anchorage system on the first post-trauma day. On the third post-trauma day, vertical ramus osteotomy, miniplate fixation of the fractured condylar neck, and free grafting were performed. When the wire was changed to a rubber band at 5 weeks postoperatively, he started smoking (half-pack a day). At 7 weeks postoperatively, the skeletal anchorage system was removed and some absorption of the condylar head was observed. At 3 months postoperatively, his mouth opening was 24 mm and no malocclusion was present, although the condylar head was distorted and malunion was observed. At 4 months postoperatively, his mouth opening was 30 mm but he complained of pain on do so. Distortion of the condylar head was aggravated. At 5 months postoperatively, his pain continued but was endurable. He continued smoking (half-pack a day) since 5 weeks postoperatively. **Conclusion:** In smokers, a longer period of immobilization is needed in bone grafting of the fractured condylar head. Longer immobilization provides sufficient time for healing and prevents smoking, since the patient cannot smoke easily when the intermaxillary fixation is applied.

Nicotine negatively impacts bone healing.^1^ In mandibular angle fractures, active smoking has a significant effect on the major complication rate (odds ratio = 4.04; *P* = .04).^2^

In high condylar fractures, we have used vertical ramus osteotomy, miniplate fixation of the fractured condylar neck, and free grafting.^3^ Usually, smoking is strictly restricted from the time of the injury to 1 month postoperatively.

We experienced a case of malunion of a condylar fracture after miniplate fixation in a patient with a 40 pack-year smoking history who restarted smoking at 5 weeks postoperatively.

## CASE

A 64-year-old man lost consciousness and fell on the ground while working in a warm workplace. Upon falling down, he hit his chin on the floor.

**Figure 1:**
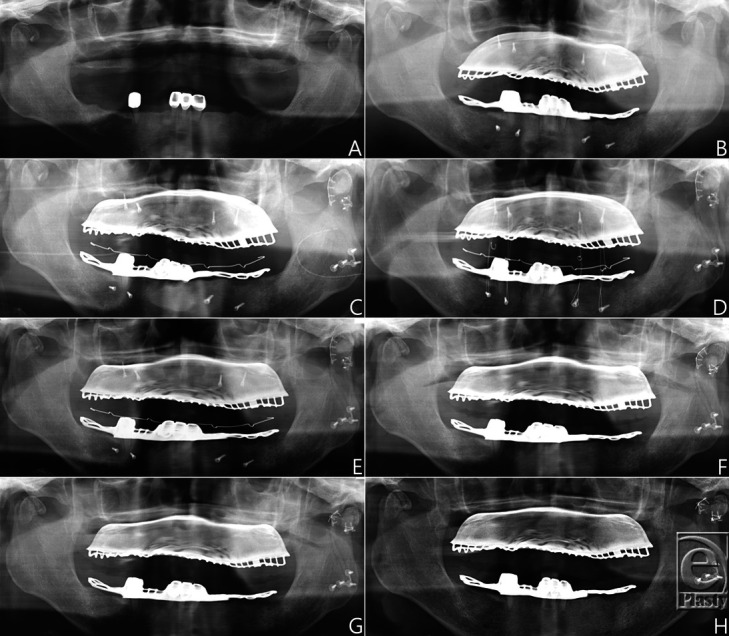
Panoramic view of the patient. (*a*) Bilateral condylar fracture and fracture of the parasymphysis. (*b*) Intermaxillary fixation was done using a skeletal anchorage system on the first PTD. (*c*) On PTD 3, vertical ramus osteotomy, miniplate fixation of the fractured condylar neck, and free grafting were performed. (*d*) The wire was changed to a rubber band at 5 weeks postoperatively. (*e*) At 7 weeks postoperatively, some absorption of the condylar head was observed. (*f*) At 3 months postoperatively, a distorted condylar head and malunion were observed. (*g*) At 4 months postoperatively, the condylar head was distorted and malunion was aggravated. (*h*) At 5 months postoperatively, the condylar head was distorted and malunion was similar at 4 months postoperatively. PTD indicates post-trauma day.

Upon examination, he had malocclusion and open bite bilaterally. The mouth opening was 1.5-finger breadths. He had a 40 pack-year smoking history. Radiology revealed a bilateral condylar fracture and a fracture of the parasymphysis (Fig 1*a*).

Intermaxillary fixation was done using a skeletal anchorage system (SAS) on the first post-trauma day (Fig 1*b*), which made it difficult for him to smoke.

**Figure 2:**
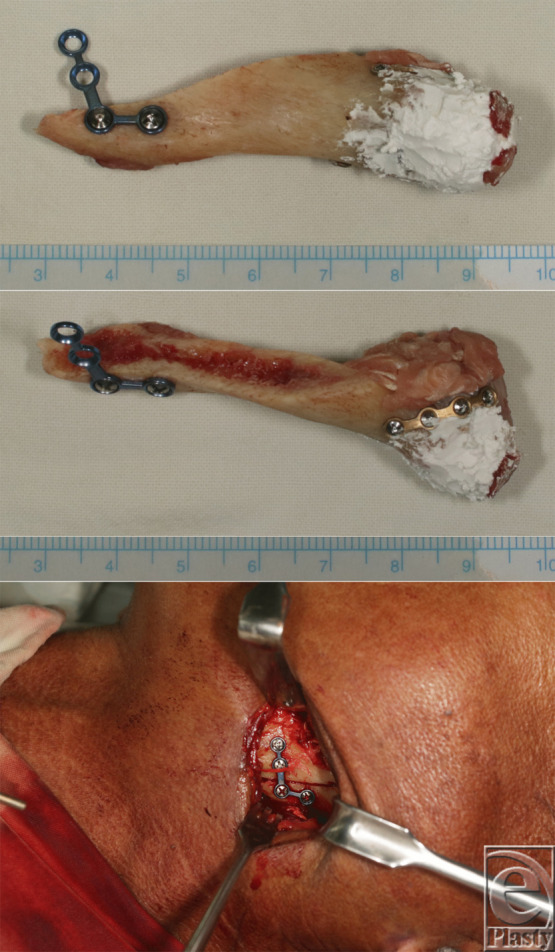
Intraoperative photographs. Vertical ramus osteotomy, miniplate fixation of the fractured condylar neck (*top*, *middle*), and free grafting (*bottom*) were performed. The defect was filled with tricalcium phosphate.

On the third post-trauma day, vertical ramus osteotomy, miniplate fixation of the fractured condylar neck, and free grafting were performed (Figs 1*c* and 2). Defect was filled with tricalcium phosphate or Ca_3_(PO_4_)_2_ (Polybone; Kyungwon Medical Co, Cheongju-si, Korea). His course in the hospital was uneventful, and he was discharged on the seventh postoperative day.

When the wire was changed to a rubber band at 5 weeks postoperatively (Fig 1*d*), he started smoking (a half-pack a day) against the medical advice. Starting at 6 weeks postoperatively, the rubber band was applied only at night.

At 7 weeks postoperatively, the SAS was removed but some absorption of the condylar head was observed in panoramic imaging (Fig 1*e*).

At 3 months postoperatively, his mouth opening was 24 mm and he showed no malocclusion. However, the condylar head was distorted and malunion was observed in a panoramic view (Fig 1*f*).

At 4 months postoperatively, his mouth opening was 30 mm and he showed no malocclusion, but he complained of pain on mouth opening. The condylar head was distorted and malunion was aggravated in a panoramic view (Fig 1*g*).

At 5 and 7 months postoperatively, his mouth opening was 30 mm and he showed no malocclusion; his pain on mouth opening continued but was endurable. He continued smoking (a half-pack a day) since 5 weeks postoperatively. At 5 months, the condylar head was distorted and malunion was similar in a panoramic view to the findings at 4 months postoperatively (Fig 1*h*).

The principles outlined in the Declaration of Helsinki were followed in this study.

## DISCUSSION

Our method (vertical ramus osteotomy, miniplate fixation of the fractured condylar neck, and free grafting) is a type of osteotomy-osteosynthesis, as described by previous authors.^4^^-^6

When totally removing the fractured condyle and placing some hydroxyapatite between the bony gaps, the following 2 things occur: remove it from its blood supply, and prevent revascularization once replaced.

Debates continue regarding the effect of smoking on dental implants. Lindfors et al^7^ described the effect of smoking on guided bone regeneration with an autogenous bone graft and concluded that smoking was associated with poor treatment outcomes. Shibli et al^8^ evaluated the effect of smoking on bone-to-implant contact and observed no osseointegration in 3 of 11 smokers. They insisted that smoking has a detrimental effect on the early bone tissue response around oxidized implant surfaces.

On the contrary, Chambrone et al,^9^ in their meta-analysis reported that the detrimental effect of smoking was not confirmed when only prospective data were assessed. Peleg et al^10^ analyzed the survival rates of sinus floor augmentation with simultaneous implant placement and stated that there were no statistically significant differences in implant failure rates between smokers and nonsmokers.

**Figure 3:**
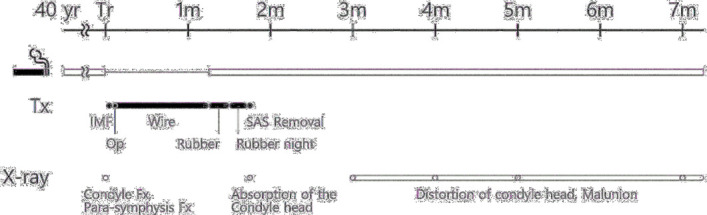
Smoking history and hospital course. First row: Time, yr: year, m: month. Second row: Smoking. Third row: Treatment (Tx), IMF: intermaxillary fixation, Op: operation, SAS: skeletal anchorage system. Forth row: x-ray findings, Fx: fracture.

In the present case, we observed that the condylar head was in the glenoid fossa until 5 weeks postoperatively (the nonsmoking period), but 2 weeks after the patient started to smoke, the grafted condylar head started to be absorbed (Fig 3). It is thought that 5 weeks is inadequate in situations where the bones have not been devascularized by removing them from the fossa or capsule.

Since smoking decreases the alveolar oxygen pressure and subcutaneous wound-tissue oxygen, and nicotine causes vasoconstriction, smokers are more likely to experience flap loss, hematoma, or fat necrosis than nonsmokers (a phenomenon known as smoking flap).^11^ As with flap surgery, in smokers, a longer period of immobilization is needed in bone grafting of a fractured condylar head. Longer immobilization provides sufficient time for healing and prevents smoking, since the patient cannot smoke easily when the intermaxillary fixation is applied.
